# Cardiology providers’ recommendations for treatments and use of patient decision aids for multivessel coronary artery disease

**DOI:** 10.1186/s12872-021-02223-y

**Published:** 2021-08-27

**Authors:** Elizabeth L. Nichols, Glyn Elwyn, Anthony DiScipio, Mandeep S. Sidhu, A. James O’Malley, Daniel D. Matlock, Shama Alam, Cathy S. Ross, Megan Coylewright, David J. Malenka, Jeremiah R. Brown

**Affiliations:** 1 The Dartmouth Institute of Health Policy and Clinical Practice, Geisel School of Medicine at Dartmouth College, 1 Medical Center Dr, Lebanon, NH 03756 USA; 2 Dartmouth-Hitchcock Medical Center, Lebanon, NH USA; 3 Albany Medical Center, Albany, NY USA; 4 Albany Medical College, Albany, NY USA; 5 University of Colorado School of Medicine, Aurora, CO USA

## Abstract

**Background:**

Rates of recommending percutaneous coronary intervention (PCI) and coronary artery bypass grafting (CABG) vary across clinicians. Whether clinicians agree on preferred treatment options for multivessel coronary artery disease patients has not been well studied.

**Methods and results:**

We distributed a survey to 104 clinicians from the Northern New England Cardiovascular Study Group through email and at a regional meeting with 88 (84.6%) responses. The survey described three clinical vignettes of multivessel coronary artery disease patients. For each patient vignette participants selected appropriate treatment options and whether they would use a patient decision aid. The likelihood of choosing PCI only or PCI/CABG over CABG only was modeled using a multinomial regression. Across all vignettes, participants selected CABG only as an appropriate treatment option 24.2% of the time, PCI only 25.4% of the time, and both CABG or PCI as appropriate treatment options 50.4% of the time. Surgeons were less likely to choose PCI over CABG (RR 0.14, 95% CI 0.03, 0.59) or both treatments over CABG only (RR 0.10, 95% CI 0.03, 0.34) relative to cardiologists. Overall, 65% of participants responded they would use a patient decision aid with each vignette.

**Conclusions:**

There is a lack of consensus on the appropriate treatment options across cardiologists and surgeons for patients with multivessel coronary artery disease. Treatment choice is influenced by both patient characteristics and clinician specialty.

**Supplementary Information:**

The online version contains supplementary material available at 10.1186/s12872-021-02223-y.

## Introduction

The ratio of percutaneous coronary intervention (PCI) to coronary artery bypass grafting (CABG) varies between hospitals across the United States and Canada [1, 2]. Coronary anatomy, indication, hospital culture, clinician recommendation, and availability of procedures influence whether a patient receives PCI or CABG [1]. Additionally clinician recommendation influences the PCI to CABG ratio at institutions [1]. Although prior studies demonstrate clinicians have different rates of recommending treatment options for coronary artery disease, we do not know whether clinicians agree on the treatment options that could be considered appropriate options for patients.

Differences among clinicians’ treatment strategy choices have previously been studied using surveys with clinical vignettes [3, 4]. Vignette surveys have been used to examine variation in physicians’ diagnosis of coronary artery disease, recommendations for self-management, and antibiotic prescribing practices [5–7]. In France, cardiologists answered questions about two clinical vignettes to determine the practice patterns for stable coronary artery disease management [8]. Clinicians varied in their diagnostic test strategies, with 24% immediately requesting coronary angiography, 49% requesting stress testing, and 27% using medial therapy without further diagnostic testing for recurrent stable angina patients [8]. A Dutch study used vignettes to assess which clinical factors, such as troponins or renal function, cardiologists prioritized when deciding whether to perform coronary angiography in patients with suspected NSTEMI [9]. There is not, to our knowledge, a vignette study assessing which treatment options clinicians consider appropriate for multivessel coronary artery disease patients.

Shared decision making is recommended for patients where treatment options offer different pros and cons, and where there may be more than one appropriate treatment. Previous shared decision making implementation projects have documented low agreement among healthcare professionals in the appropriate approach to shared decision making [10]. A review of five encounter decision aid trials showed variability in how and when clinicians used decision aids [11]. Past studies have shown clinicians are more likely to support shared decision making when there is no strong preference for one treatment option. Support for shared decision making is reduced when a specific treatment option is favored [12]. It is important to determine whether clinicians consider more than one treatment option appropriate in a clinical scenario when proposing a patient decision aid. In multivessel disease, using a clinical vignette study allows us to additionally study clinicians’ willingness to use a patient decision aid.

Understanding whether clinicians agree on the appropriate treatment options helps us determine the drivers of variation in the PCI to CABG ratios and clinicians’ willingness to use patient decision aids. The primary aim of this study was to assess differences between clinicians on the appropriate treatment options for multivessel coronary artery disease patients, in addition to examining clinicians’ willingness to use patient decision aids. This was evaluated through a survey of clinicians in Northern New England Cardiovascular Disease Study Group. The survey asked about clinicians’ treatment recommendations and willingness to use a patient decision aid in differing clinical scenarios.

## Methods

### Participants and setting

This is a cross sectional study of Northern New England Cardiovascular Study Group (NNECDSG) regular meeting participants. The NNECDSG includes clinical cardiologists and interventional cardiologists, cardiothoracic surgeons, health services researchers, nurses, technicians, and other healthcare providers who work in cardiovascular care and have attended at least one of the Northern New England Meetings in the past 2 years. The survey was administered first at an NNECDSG meeting in October 2015, where all meeting attendees completed the survey. NNECDSG regular meeting participants who did not attend the October meeting received an email version of the survey in July 2016. Emailed survey responses were accepted through December 2016.

### Survey development

The survey, available in the Data Supplement was developed by health services researchers at The Dartmouth Institute and NNECDSG and informed by a literature review of factors that influence the choice between CABG and PCI. The survey included questions about demographics, attitudes towards patient decision support tools, and questions about treatment recommendations for each of the three clinical vignettes. The attitude toward patient decision support tools was determined using a question that asks participants to select words from a provided list that describe utilizing patient decision support tools. The adjective list includes 10 positive adjectives such as worthwhile and rewarding, and 10 negative adjectives such as difficult and inconvenient (Additional file 1).

Vignettes are short case histories of theoretical patients based on real clinic scenarios [3]. Members of the research team drafted each clinical vignette based on the characteristics of a pair of matched patients. Matched patients found in the NNECDSG registry who had similar anatomical characteristics and comorbidities, but had received different procedures provided the characteristics of patients in each the vignettes. This was done to ensure each patient in the vignettes could theoretically be eligible for either CABG or PCI, despite each vignette having different patient characteristics. We ensured each vignette had different characteristics that physicians may weigh differently when choosing treatment strategies. The first vignette is male, 69 years old, and has 80% left anterior descending artery (LAD), 65% circumflex, 90% right coronary artery (RCA). The second vignette describes an 81 year-old woman who is diabetic with 80% LAD, 35% diagonal, and 75% circumflex stenosis, while the third patient is a 63 year-old male with unstable angina and an 85% LAD, 60% circumflex, and 75% RCA stenosis. Alternating characteristics of age, diabetes, priority, and anatomical characteristics gave variation to the patients that might elucidate how physicians choose different treatment strategies that optimally match patient characteristics. To ensure all vignettes could be considered eligible for multiple treatment options, each vignette patient had normal ventricular function and no significant valvular heart disease. Three clinicians, including a cardiologist and a surgeon, reviewed each clinical vignette to ensure the vignettes were realistic and patients could be considered eligible for PCI or CABG as an appropriate treatment option. After being clinically reviewed the vignettes were edited and redistributed to clinician reviewers to ensure any issues were resolved. The vignettes and a short description of patient characteristics are displayed in Table 1.

**Table 1:** Clinical vignette patients and their characteristics

	Vignette	Characteristics
Smith	*“Mr. Smith is a 69-year-old with class III stable angina. He first noticed his chest was tight during his daily swim at the rec center a year ago. After his sister prodded him, he went to the doctor and tried out different medications. He had a lot of side effects and struggled to find medications that worked for him. He settled into taking aspirin and a beta blocker and most of the time takes a calcium channel blocker. He still gets chest pain sometimes and avoids one of his favorite hunting spots because there’s a big hill. After discussing his lingering symptoms and disappointment with medications with his cardiologist, he is scheduled for a stress test. His stress test shows evidence of ischemia and his ECHO shows normal ventricular function and no valve disease. He undergoes a cath. At his cath they find an 80% LAD stenosis, 65% circumflex stenosis, and 90% RCA stenosis.”*	Male 69 years old Rx: aspirin, beta blocker, calcium channel blocker 80% LAD, 65% circumflex, 90% RCA
Adams	“*Mrs. Adams is 81 years old. She is diabetic and her hemoglobin A1c has been regularly under 7.2% for years. In addition to her diabetes medication, she takes aspirin, a statin, and a beta blocker. She has class III stable angina and is mainly bothered by her chest pain when she is working in her garden. Although her symptoms haven’t changed recently, her cousin had a heart attack last month and at her check-up with her cardiologist she asked if she could do something more for her heart. She has normal ventricular function and no valve disease. She and her cardiologist decide it is time to proceed with a cath and she is scheduled for the next week. During a diagnostic cath, they find an 80% LAD stenosis, a 30% diagonal stenosis, and 75% circumflex stenosis.”*	Female 81 years old Diabetic Rx: aspirin, statin, beta blocker, 80% LAD, 35% diagonal, 75% circumflex
Jones	“*Mr. Jones is a 63-year-old male. He has been managed medically for stable angina for 3 years. His wife makes sure he takes his aspirin, an ACE inhibitor, and a beta blocker but lately feels like his medications don’t do as much as they used to. He is not diabetic. Two days ago his chest pain got worse than normal while on his typical evening walk with his wife. His chest pain didn’t go away as quickly as usual and lingered through the morning when they decided to go to the hospital. After he was admitted he had an ECG. It showed no ST or TW changes and his troponins came back normal. He is scheduled for a cath the following morning. By the next morning his chest pain is not bothering him anymore, but he and his wife are concerned enough about his symptoms that they want to proceed with a cardiac cath. The cath shows an 85% LAD stenosis, a 60% circumflex stenosis, and a 75% RCA stenosis. His LV gram shows normal ventricular function and no valve disease.*”	Male 63 years old Rx: aspirin, ACE inhibitor, beta blocker Unstable angina 85% LAD, 60% circumflex, 75% RCA

For each clinical vignette, the survey asked “Which treatments are appropriate options for this patient? Check all that apply.” The possible responses were CABG, PCI, ad-hoc PCI, and medical therapy only. We also asked “Would you use the Multi-vessel Coronary Artery Disease Comparison Table with this patient?” for each clinical vignette, with possible answers of yes or no. The Multivessel Coronary Artery Disease Comparison Table is a one-page clinical encounter patient decision aid developed with the input of NNECDSG members and researchers over the previous year. NNECDSG members vetted evidence, reviewed language, and gave feedback on the usability of the patient decision support tool. Participants that responded “No” to using the patient decision aid were asked “If no, what are your main reasons? Check all that apply.” Response options included: “(1) there is only one appropriate treatment option (2) the information in the patient decision aid is not applicable to this patient because *[fill in the blank]* (3) I don’t think the patient would be able to grasp the nuances of the decision (4) I need more information (i.e. FFR, stress test) to choose the right treatment for the patient. I would need to know *[fill in the blank] (5)* other: *[fill in the blank]*.”

### Administration

During the plenary session at the NNECDSG envelopes containing the information sheet, survey, and a blank envelope were distributed to all attendees (55 members). Members were asked to complete the enclosed survey, seal it in the blank envelope, and return the survey. Responses were kept anonymous, however we maintained a list of all participating members who attended the meeting so we could target non-attendees with an online survey. Survey collection continued throughout the day with additional announcements to encourage survey completion during morning, lunch, and afternoon breaks. Meeting survey data was entered into a standardized electronic data collection form and independently checked by another research assistant. We used the NNECDSG email registration list to identify NNECDSG cardiologists and cardiothoracic surgeons who did not attend the 2015 October meeting. These additional NNECDSG members were emailed a link containing the survey built within Qualtrics (version 12.2016, Qualtrics, UT). The email was sent to 49 participants on July 9th 2016 with follow up email reminders every two weeks to remaining non-responders through December 2016.

### Data analysis

The attitude towards patient decision support tools was determined as positive, neutral, or negative based on the net positive or negative adjectives selected. We described the treatment options chosen and willingness to use the patient decision aid for each vignette. We categorized participants as surgeons, cardiologists, nurses, or other. Cardiologists include both clinical and interventional cardiologists, nurses include nurses and nurse practitioners. The other category includes perfusionists, anesthesiologists, researchers, administrators, and data analysts as displayed in Table 2. All participants making up the other category were grouped together because they are not directly responsible for making treatment decisions with multivessel coronary artery disease patients.

**Table 2:** Participant type characteristics

Participant type	Occupation
Cardiologists	Interventional cardiologists
	Invasive cardiologists
	General cardiologists
Surgeons	Cardiothoracic surgeons
Nurses	Nurses
	Nurse practitioners
Other	Perfusionists
	Anesthesiologists
	Researchers
	Data analysts
	Administrators

The treatment choice for each vignette was organized into the final categories: CABG, PCI, or both. CABG only reflected participants who chose only CABG, or CABG and medical therapy. The PCI only category includes the responses PCI alone, PCI and ad-hoc PCI, PCI and medical therapy, PCI ad-hoc PCI and medical therapy, or ad-hoc PCI and medical therapy. Ad-hoc PCI was always counted as a PCI option. The both category consisted of any combination of CABG and PCI or ad-hoc PCI such as CABG or PCI, CABG PCI or medical therapy, CABG PCI or ad-hoc PCI, CABG PCI ad-hoc PCI or medical therapy, and CABG or ad-hoc PCI. Medical therapy was selected alone 5 times (1.9% of all vignette responses) and was counted as missing when it was the only selected treatment; otherwise the response was categorized with whichever other treatments were selected.

Treatment selections were compared across participant type using a generalized Cochran-Mantel–Haenszel test for association between participant type and treatment choice stratified by vignette. We then used a multinomial regression model of treatment choice with predictors indicating vignette and participant type and inflating standard errors for the clusters of observations within participants. We considered including in the model the participant’s age, training length, center’s use of patient decision aids, mode of delivery, and attitude towards patient decision aids, but none changed the overall point estimate. We tested for an interaction between participant type and vignette, but did not find a significant interaction. CABG was chosen as the base outcome because it is considered the most clinically conservative treatment method. We used a logistic regression of patient decision aid use by vignette and provider type with random effects by participant.

## Results

Across both distribution modes, 88 (84.6%) NNECDSG members responded, 55 (100%) of meeting attendees and 33 (67.3%) members receiving emails. Participant characteristics are described in Table 3. Most participants were cardiologists (33, 37.5%), followed by nurses (19, 21.6%) others (19, 21.6%) and then cardiothoracic surgeons (17, 19.3%). Twenty-four (27.3%) participants had been out of training for 20–29 years, and 20 (22.7%) had been out of training for 30–39 years. Only 40 participants (48.8%) reported their center used patient decision aids. Most participants had a positive attitude about patient decision aids (64, 72.7%).

**Table 3:** Participant characteristics

	N	%
Meeting responder	55	62.5
Online responder	33	37.5
Participant type		
Cardiologists	33	37.5
Cardiothoracic surgeons	17	19.3
Nurses	19	21.6
Other	19	21.6
Age, years		
18–29	2	2.3
30–39	10	11.4
40–49	20	22.7
50–59	31	35.2
60–69	23	26.1
70+	2	2.3
Years since training		
0–9	13	14.8
10–19	15	17.1
20–29	24	27.3
30–39	20	22.7
40–49	2	2.3
Still in	2	2.3
NA	12	13.6
Center with patient decision aids	40	48.8
Attitude towards patient decision aids		
Positive	64	72.7
Neutral	11	12.5
Negative	13	17.8

Treatment choice varied by clinical vignette, for example when comparing vignette 2 to vignette 1, participants were more likely to choose PCI only over CABG only (RR 11.13, 95% CI 3.96, 31.28) and both treatments over CABG only (RR 3.23, 95% CI 1.23, 8.46).

### Clinical vignette 1: Mr. Smith

**Fig. 1:**
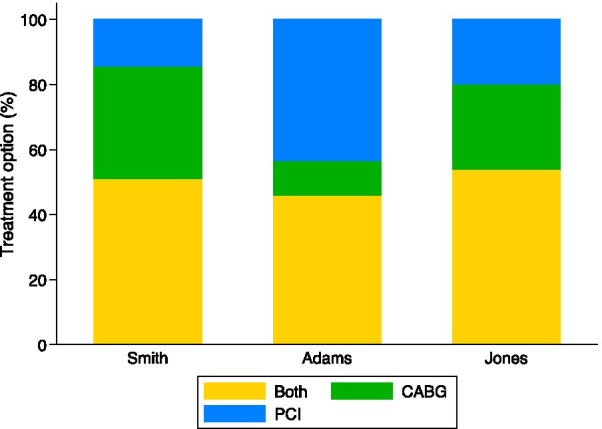
Percent of participants that selected coronary artery bypass grafting (CABG), percutaneous coronary intervention (PCI) or both treatments for each vignette. Treatment selection varied by vignette

**Fig. 2:**
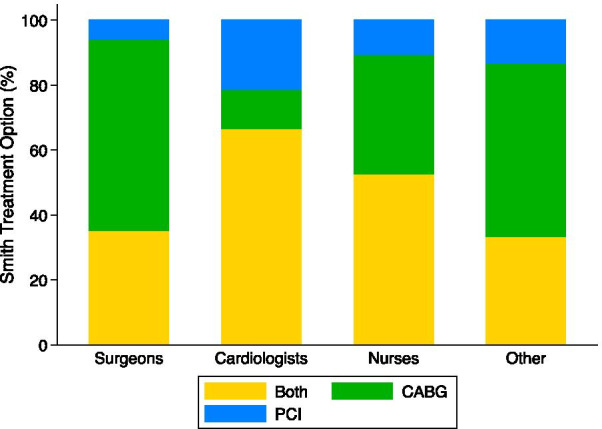
Percent of participants that selected coronary artery bypass grafting (CABG), percutaneous coronary intervention (PCI) or both treatments by participant type for the Smith vignette (69 year old male, 80% LAD, 65% circumflex, and 90% RCA stenosis)

The Smith clinical vignette describes a 69-year-old male with 80% LAD stenosis, 65% circumflex stenosis, and 90% RCA stenosis (Table 1). In the Smith vignette, 39 (34.5%) chose CABG, 12 (14.3%) chose PCI, and 43 (51.2%) chose both (Table 4, Fig. 1). The distribution of treatment choices across participant types is displayed in Table 4 and Fig. 2. Surgeons (10, 58.8%) and other participants (8, 53.3%) selected CABG most frequently, while 22 (66.7%) cardiologists and 10 (52.6%) nurses selected a combination of both treatments most frequently.

**Table 4:** Participant responses to ‘*Which treatments are appropriate options for this patient? Check all that apply*

	Overall		Surgeon		Cardiologists		Nurses		Other	
	N	(%)	N	(%)	N	(%)	N	(%)	N	(%)
Smith										
CABG	29	(34.5)	10	(58.8)	4	(12.1)	7	(36.8)	8	(53.3)
PCI	12	(14.3)	1	(5.9)	7	(21.2)	2	(10.5)	2	(13.3)
Both	43	(51.2)	6	(35.3)	22	(66.7)	10	(52.6)	5	(33.3)
Adams										
CABG	8	(10.5)	2	(12.5)	2	(6.5)	1	(6.7)	3	(21.4)
PCI	33	(43.4)	8	(50.0)	7	(22.6)	10	(66.7)	8	(57.1)
Both	35	(46.1)	6	(37.5)	22	(71.0)	4	(26.7)	3	(21.4)
Jones										
CABG	20	(26.3)	8	(47.1)	1	(3.5)	6	(40.0)	5	(33.3)
PCI	15	(19.7)	0	(0.0)	6	(20.7)	5	(33.3)	4	(26.7)
Both	41	(54.0)	9	(52.9)	22	(75.9)	4	(26.7)	6	(40.0)

Of coronary artery bypass grafting (CABG), percutaneous coronary intervention (PCI), or both by vignette and participant type

### Clinical vignette 2: Mrs. Adams

**Fig. 3:**
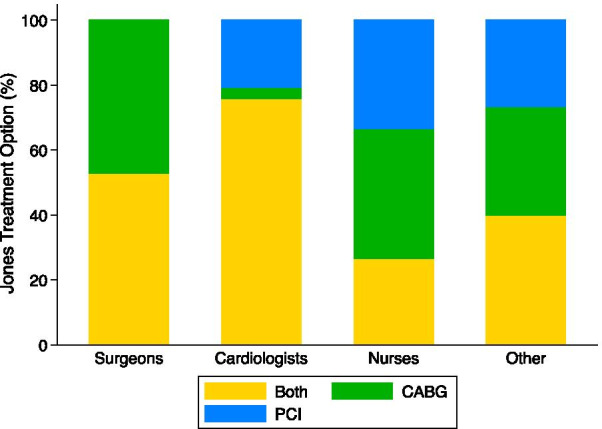
Percent of participants that selected coronary artery bypass grafting (CABG), percutaneous coronary intervention (PCI) or both treatments by participant type for the Jones vignette (63 year old male with unstable angina and 85% LAD, 60% circumflex, and 75% RCA stenosis)

The Adams vignette describes an 81-year-old diabetic female with 80% LAD stenosis, 35% diagonal stenosis, and 75% circumflex stenosis (Table 1). In the Adams vignette, CABG was chosen as an appropriate treatment option by 8 (10.5%) participants, PCI by 33 (43.4%) participants, and both treatments by 35 (46.1%) participants (Table 4, Fig. 1). The distribution of treatment choices by participant types is displayed in Table 4 and Fig. 3. PCI was selected most frequently by all participant types except cardiologists, of whom 22 (71.0%) chose both treatments. PCI was chosen by 8 (50.0%) surgeons, 10 (66.7%) nurses, and 8 (57.1%) other participants.

### Clinical vignette 3: Mr. Jones

**Fig. 4:**
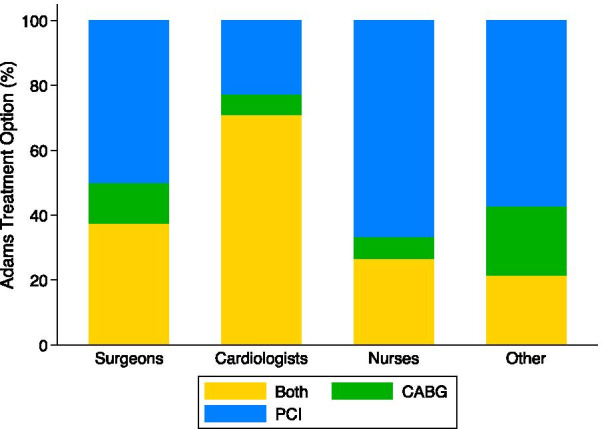
Percent of participants that selected coronary artery bypass grafting (CABG), percutaneous coronary intervention (PCI) or both treatments by participant type for the Adams vignette (81 year old female, diabetic with 80% LAD, 35% diagonal, 75% circumflex stenosis)

The Jones vignette describes a 63-year old male with unstable angina and a 85% LAD stenosis, 60% circumflex stenosis, and 75% RCA stenosis. For the Jones vignette, 20 (26.3%) participants chose CABG, 15 (19.7%) chose PCI, and 41 (54.0%) chose both treatments as appropriate (Table 4). Table 4 and Fig. 4 show the distribution of treatment choices by participant type. Both treatment options were most frequently selected with 9 (52.9%) surgeons, 22 (75.9%) cardiologists, and 6 (40.0%) other participants. Nurses selected CABG most frequently (6, 40.0%).

### Treatment choice

Across all vignettes, participants chose CABG only 24.2% of the time, PCI only 25.4% of the time, and both treatment options 50.4% of the time. Therefore 49.6% of the time, participants chose only one treatment as an appropriate option. Overall, 19 (21.6%) participants chose only one treatment option for each vignette. A generalized Cochran-Mantel–Haenszel test showed an association between treatment choice of CABG, PCI, or both and participant type accounting for vignette (p < 0.0001). Table 4 shows the results of a multinomial regression for treatment choice. The clinical vignette significantly changed treatment choice. For vignette 2 (Adams) compared to vignette 1 (Smith), participants were significantly more likely to choose PCI relative to CABG (RR 11.13, 95% CI 3.96, 31.28) and both treatments relative to CABG (RR 3.23, 95% CI 1.23, 8.46). In vignette 3 (Jones) compared to vignette 1 (Smith) there was no difference in likelihood of choosing PCI over CABG (RR 1.97 95% CI 0.99, 3.90) or choosing both treatments over CABG (1.52, 95% CI 0.93, 2.51).

Treatment choice was also significantly associated with participant type. Surgeons were less likely to choose PCI over CABG (RR 0.14, 95% CI 0.03, 0.59) or both treatments over CABG (RR 0.10, 95% CI 0.03, 0.34) relative to cardiologists. There was no significant difference in choosing PCI over CABG between nurses (RR 0.45, 95% CI 0.10, 1.98) or other participants (RR 0.29, 95% CI 0.07, 1.19) compared to cardiologists. However the likelihood of choosing both treatments over CABG was significantly lower in nurses (RR 0.14, 95% CI 0.04, 0.50) and other participants (RR 0.09, 95% CI 0.03, 0.32) compared to cardiologists, making cardiologists the most likely participant type to choose both treatments over CABG.

### Patient decision aid use

Forty-seven participants (65.3%) would use the patient decision aid in every vignette and 8 participants (13.6%) said they would not use the decision aid in any vignette (Table 5). Sixty-two participants (73.8%) responded they would use the patient decision aid with the patient described in the vignette 1 (Smith). The most common reason for not using the patient decision aid was ‘Other’ (11, 12.5%) and included the patient decision aid being too complicated or a disagreement with the data in the patient decision aid. The second most frequently selected reason for not using the patient decision aid was ‘there is only one appropriate treatment option’ selected by 7 (7.95%) participants. In vignette 2 (Adams), 65 participants (80.3%) responded they would use the patient decision aid with the patient. The most common reason for not using the patient decision aid selected by 6 (9.7%) participants was needing more information, specifically fractional flow reserve. In vignette 3 (Jones), 57 participants (73.1%) responded that they would use the patient decision aid with the patient. The most common reason for not using the patient decision aid was ‘other’ selected by 7 (7.95%) participants, often because the risks for the patient were not represented accurately and participants felt the risks led to one option being the best for the patient. The second most common reason for not using the patient decision aid was ‘there is only one appropriate treatment option’ selected by 6 (6.8%) participants.

**Table 5:** Multinomial regression of treatment choice of percutaneous coronary intervention (PCI), or both PCI and coronary artery bypass grafting (CABG) compared to CABG as the reference group

	Univariate Model					Full Model			
CABG (ref)	PCI		Both		PCI			Both	
	RR	(95% CI)	RR	(95% CI)	RR		(95% CI)	RR	(95% CI)
Vignette									
Smith	1	ref	1	ref	1		ref	1	ref
Adams	9.97	(3.75, 26.49)	2.95	(1.29, 6.73)	11.13		(3.96, 31.28)	3.23	(1.23, 8.46)
Jones	1.81	(0.93, 3.52)	1.38	(0.90, 2.13)	1.97		(0.99, 3.90)	1.52	(0.93, 2.51)
Participant type									
Cardiologist	1	ref	1	ref	1		ref	1	ref
Surgeon	0.16	(0.04, 0.58)	0.11	(0.04, 0.35)	0.14		(0.03, 0.59)	0.10	(0.03, 0.34)
Nurse	0.43	(0.11, 1.59)	0.14	(0.04, 0.47)	0.45		(0.10, 1.98)	0.14	(0.04, 0.50)
Other	0.31	(0.09, 1.05)	0.09	(0.03, 0.32)	0.29		(0.07, 1.19)	0.09	(0.03, 0.32)

Relative risk (RR) reflects the relative likelihood of choosing PCI or both treatments over CABG, compared to that of the referent group in each variable

In a logistic model of patient decision aid use, we included vignette, treatment choice, participant type. There was no significant difference in decision aid use in vignette 2 (Adams) (OR: 2.47, 95% CI 0.76, 8.06) or vignette 3 (Jones) (OR: 1.03, 95% CI 0.34, 2.77) relative to vignette 1 (Smith). Participants were more likely to use the patient decision aid when they had selected both treatments as appropriate (OR: 5.94, 95% CI 1.04, 33.98) compared to when they selected CABG only. There was no significant difference in decision aid use when they selected PCI only compared to CABG only (OR: 1.55, 95% CI 0.19, 12.60). There was no significant difference in use of patient decision aid in surgeons (OR: 1.36, 95% CI 0.16, 11.59), nurses (OR: 22.60, 95% CI 0.89, 575.66), or other participants (OR: 4.59, 95% CI 0.34, 61.12) compared to cardiologists.

## Discussion

Participant’s opinions of appropriate treatment options varied based on vignette and participant specialty. Both PCI and CABG options were chosen together as appropriate treatments only 50.4% of the time, and the remaining 49.6% of the time only one treatment option was chosen as appropriate. Participants were more likely to select PCI or both treatments as appropriate over CABG for the Adams vignette relative to the Smith vignette. Cardiologists were the most likely participant type to choose both treatments over CABG alone. Surgeons were the only participant specialty significantly less likely to choose PCI over CABG compared to cardiologists. Overall, 65% of participants thought they would use the patient decision aid with every vignette, even though 53.2% of these participants chose only one treatment option in at least one of the clinical vignettes. Patient decision support tool use was more likely when both CABG and PCI were chosen as appropriate treatment options, but was not influenced by vignette or participant type.

Past studies have shown differences among healthcare provider type in care recommendations [13]. McIlvennan described variation in cardiologists’ and hospice/palliative medicine clinicians’ views of left ventricular assist device deactivation [14]. This included their approach and comfort with deactivation of left ventricular assist devices [14]. In a survey of French cardiologists looking at care management choices for stable coronary artery disease patient vignettes, Bauters and colleagues found practice patterns in even routine clinical situations such as β-blocker prescription, or management for recurrent chest pain after revascularization vary when there are no high-level practice guidelines [8].

In observational studies, variation in revascularization rates have been attributed to patient factors, similar to our finding that treatment selections change based on clinical vignettes [15]. An analysis of Ontario patients with stable ischemic heart disease showed 67.4% of the variation in revascularization strategies was due to patient factors [15]. However, when examining use of PCI and CABG across Ontario and New York State, rates of PCI were higher in New York, most likely related to the different reimbursement approaches in Ontario and New York [2]. Other studies have documented hospital ‘cultural’ effects, and physician type impacting revascularization strategy, in addition to patient factors [1].

This study has a number of limitations, which must be considered while interpreting results. The surveys were completed by a small sample of clinicians in northern New England, and their opinions may be influenced by the local culture of practice. The vignettes could not feasibly include all potentially relevant clinical details that may affect treatment choice. Additionally the vignettes did not include explicit patient preferences, which could also impact the treatment choice selected by the participant. To more closely examine which clinical factors influence treatment selection, vignettes with systematically varied clinical factors could be used in a combination with prediction models from registries [6, 16]. Other system factors such as reimbursement structure, hospital culture, volume and availability or procedures also could not be accounted for in this study.

One next step to validate these findings and further explore the variation in provider preferences for treatment of multivessel disease, is to administer this survey at a national level with the NCDR and STS jointly. The inclusion of cardiac catheterization images or video and inclusion of patient preferences, occupation, medical insurance, and living situation could also help to improve the evaluation of variation in provider specialty preferences for multivessel coronary artery disease.

## Conclusions

There is a lack of consensus on the appropriate treatment options across cardiologists and surgeons for patients with multivessel coronary artery disease. Treatment choice is influenced by both patient characteristics and clinician type. Although most participants were willing to use a patient decision support tool, many participants did not consider the vignette patients eligible for more than one treatment which could affect their likelihood of consulting with other clinical team members during decision making or using a patient decision aid.

## Supplementary Information

**Additional file 1.** Survey.

## Data Availability

All data generated or analysed during this study are included in this published article (and its additional files).
